# Targeting mTOR pathway inhibits tumor growth in different molecular subtypes of triple-negative breast cancers

**DOI:** 10.18632/oncotarget.10195

**Published:** 2016-06-21

**Authors:** Rana Hatem, Rania El Botty, Sophie Chateau-Joubert, Jean-Luc Servely, Dalila Labiod, Ludmilla de Plater, Franck Assayag, Florence Coussy, Céline Callens, Sophie Vacher, Fabien Reyal, Sabina Cosulich, Véronique Diéras, Ivan Bièche, Elisabetta Marangoni

**Affiliations:** ^1^ Genetics Department, Institut Curie, PSL Research University, Paris, France; ^2^ Faculty of Pharmacy, Aleppo University, Aleppo, Syria; ^3^ Translational Research Department, Institut Curie, PSL Research University, Paris, France; ^4^ BioPôle Alfort, National Veterinary School of Alfort, Maisons Alfort, France; ^5^ INRA, PHASE Department, Paris, France; ^6^ Surgery Department, Institut Curie, PSL Research University, Paris, France; ^7^ AstraZeneca R&D Cambridge, CRUK Cambridge Institute, Cambridge, UK; ^8^ Medical Oncology Department, Institut Curie, PSL Research University, Paris, France; ^9^ EA7331, University of Paris Descartes, Paris, France

**Keywords:** TNBC, mTOR, PI3K pathway, PDX

## Abstract

Triple-negative breast cancers (TNBC) are characterized by frequent alterations in the PI3K/AKT/mTOR signaling pathway. In this study, we analyzed PI3K pathway activation in 67 patient-derived xenografts (PDX) of breast cancer and investigated the anti-tumor activity of the mTOR inhibitor everolimus in 15 TNBC PDX with different expression and mutational status of PI3K pathway markers.

Expression of the tumor suppressors PTEN and INPP4B was lost in 55% and 76% of TNBC PDX, respectively, while mutations in *PIK3CA* and *AKT1* genes were rare. In 7 PDX treatment with everolimus resulted in a tumor growth inhibition higher than 50%, while 8 models were classified as low responder or resistant. Basal-like, LAR (Luminal AR), mesenchymal and HER2-enriched tumors were present in both responder and resistant groups, suggesting that tumor response to everolimus is not restricted to a specific TNBC subtype. Analysis of treated tumors showed a correlation between tumor response and post-treatment phosphorylation of AKT, increased in responder PDX, while PI3K pathway markers at baseline were not sufficient to predict everolimus response.

In conclusion, targeting mTOR decreased tumor growth in 7 out of 15 TNBC PDX tested. Response to everolimus occurred in different TNBC subtypes and was associated with post-treatment increase of P-AKT.

## INTRODUCTION

Triple-negative breast cancer represents 15% of breast cancers and is defined by the lack of detectable expression of estrogen and progesterone receptors and HER2 amplification [1]. This subtype of breast cancer is associated to a poor prognosis, as tumor relapses are frequent in the early stage and tumor become resistant to conventional chemotherapies in the metastatic setting [2]. As TNBCs have no indications for endocrine therapy or HER2 inhibitors, novel targeted therapies are needed. Interestingly, the basal-like subgroup, which shows the greatest overlap with TNBC, is associated to the highest activation of downstream members of the PI3K signaling pathway as determined by gene expression levels and proteomic arrays [3]. Genomic aberrations observed in the PI3K pathway in basal-like tumors include loss/mutation of PTEN, loss of INPP4B, amplification/ mutation of *PIK3CA* (the gene encoding the p110 catalytic subunit of the PI3K). The PTEN and PIK3CA alterations occur early in breast tumor initiation and seem to be present in dominant tumor clones [4, 5]. As a negative regulator of the PI3K pathway, loss of PTEN function through mutational inactivation or down-regulation of expression results in activation of PI3K–AKT-mTOR signaling. More recently, Fedele et al. reported that the INPP4B protein functions as a tumor suppressor by negatively regulating epithelial cell proliferation through regulation of PI3K–AKT-mTOR pathway, and that loss of INPP4B is a marker of human basal-like carcinomas [6]. INPP4B protein loss was also frequently observed in PTEN-null tumors showing the existence of co-occurent loss of two phosphoinositide phosphatases in human breast cancer. This provides evidence for the cooperative promotion of oncogenesis through alterations to multiple components of the PI3K signaling pathway. There are currently no targeted therapies for the treatment of human basal-like cancers and tumors exhibiting loss of PTEN and/or INPP4B proteins may represent appropriate candidates for treatment with PI3K pathway inhibitors. The mammalian target of rapamycin (mTOR) is an effector of the PI3K signalling pathway regulated by AKT and the tumor-suppressor PTEN. Although the activity of the mTOR inhibitor everolimus has been reported in patients with luminal and HER2+ breast cancers [7, 8], results of clinical trials with mTOR-specific inhibitors in TNBC have not been published yet. Identification of biomarkers to help select patients who are most likely to benefit from treatment with PI3K/AKT/mTOR pathway inhibitors is an essential unmet need, and biomarker analysis is a core component of many ongoing clinical trials. In this study we used a panel of molecularly characterized PDX of TNBC to evaluate the efficacy of everolimus in tumors with different genomic alterations. We provide evidence that a subset of TNBC PDX models significantly responds to everolimus *in vivo*, including PDX models resistant to conventional chemotherapies. Response to everolimus occurred in different TNBC subtypes and was associated to post-treatment increased phosphorylation of AKT. By contrast, intrinsic phosphorylation of AKT as well as PTEN and INPP4B losses were not associated with everolimus activity.

## RESULTS

### Loss of PTEN and/or INPP4B proteins occurs at high frequency in TNBC PDX

**Figure 1:**
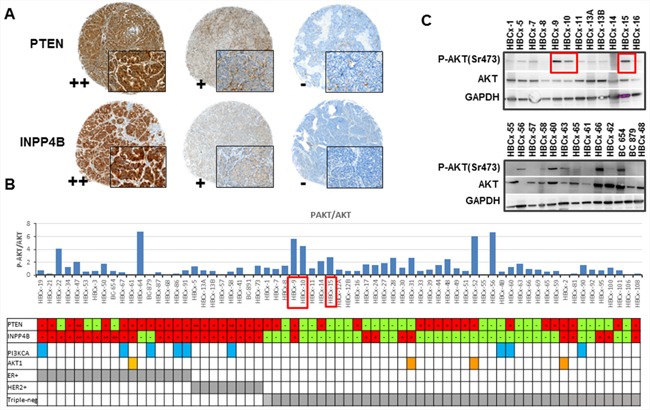
PI3K pathway status in PDX models **A.** IHC staining of PTEN and INPP4B in PDX models showing different expression intensities (++, + and -). **B.** P-AKT/AKT ratio quantified from western blot analysis of 67 PDX models. Each bar presents a single PDX. PDX models with a *PIK3CA* mutation are marked with blue squared: HBCx-19 carried the E542K mutation, HBCx-67, HBCx-86 and HBCx-4B carried the E545K mutation and BC-879, HBCx-58, HBCx-60, HBCx-90 and HBCx-91 the H1047R mutation. PDX carrying the AKT1 mutation E17K are marked with orange squared. **C.** Western blot analysis of AKT, P-AKT (Ser473) and GAPDH in 25 PDX models. Red squares in Figure 1B and 1C indicate as examples 3 PDX models with high P-AKT/AKT ratio.

To identify xenografts models with tumor suppressor loss and to determine the frequency of concomitant loss of INPP4B and PTEN proteins, we analyzed PTEN and INPP4B expression by immunohistochemistry (IHC). Sixty-seven breast cancer PDX including 42 TNBC, 17 ER+ and 8 HER2+ tumors, were stained with primary antibodies against PTEN and INPP4B. Three different scores were assigned to expression intensity (++, strong expression; + moderate expression and –, no expression) (Figure 1A). Within the triple-negative subgroup, PTEN and INPP4B protein expression were lost (−) in 55% and 76% of tumors, respectively (Table 1). Seventeen PDX models (41%) displayed a concomitant loss of both PTEN and INPP4B proteins. PTEN and INPP4B proteins were lost in 24% and 12% of ER+ tumors, respectively, and in none of the 8 HER2+ models analyzed. These results indicate that PTEN and INPP4B losses occur more frequently in TNBC than in non TNBC (p=0.002 and p<0.0001, respectively, Fisher's exact test).

**Table 1:** frequency of PTEN and INPP4B loss in PDX models of ER+, HER2+ and triple-negative (TN) breast cancer, determined by IHC analysis

Subtype	N° of PDX	PTEN loss (%)	INPP4B loss (%)	both
ER+	17	4 (24%)	2 (12%)	0
HER2+	8	0	0	0
Triple-negative	42	23 (55%)	32 (76%)	17 (41%)
Total	67	27 (41%)	34 (51%)	17 (26%)

Next, we analyzed the phosphorylation levels of AKT (ser473) and S6 (a downstream target of mTOR) by western blot. Figure 1B shows the ratio of P-AKT/AKT quantified from western blot analysis (each bar represents a different PDX model). The ratio between phosphorylated and total AKT was heterogeneous across the PDX panel, in 52% of TNBC tumors, this ratio was greater than 1. Two western blots including 25 PDX are shown as examples in Figure 1C.

By contrast, S6 was found to be phosphorylated in the great majority of tumors (data not shown).

Finally, we performed targeted sequencing of *PIK3CA* and *AKT1* hot spot mutations in the panel of PDX models (Figure 1B). Nine PDX models carried an activating *PIK3CA* mutation: 5 ER+, 1 HER2+ and 3 triple-negative tumors, 2 of them established from metaplastic breast cancers (details on *PIK3CA* mutations are provided in Figure 1 legend). One ER+ and 3 triple-negative PDX carried the E17K *AKT1* mutation.

In summary, these results indicate that the majority of TNBC xenografts show loss of one or both tumor suppressor proteins PTEN and INPP4B, activation of PI3K pathway and rare *PIK3CA* and *AKT1* mutations.

### Response to everolimus is not restricted to specific TNBC subtypes

We next addressed the question whether the genomic alterations previously identified are associated to response to mTOR inhibitors. We determined the anti-tumor activity of everolimus, an mTORC1 inhibitor approved for the treatment of metastatic ER+ breast cancers, in 15 PDX models of TNBC, whose histological and molecular characteristics are summarized in Table 2. The panel included 12 infiltrating ductal carcinomas (IDC) and 3 metaplastic breast carcinomas (MBC), two spindle (HBCx-60 and HBCx-66) and one chondroid (HBCx-69). The 15 PDX models were chosen based on different status of PI3K pathway markers (expression of PTEN, INPP4B and AKT1/PIK3CA mutations) (Table 2). The tumor genomic characteristics as well as the phosphorylation status of AKT and S6 are summarized in Table 2. Immunohistochemistry analysis of PTEN, INPP4B and P-AKT(Ser473) are shown in Supplementary Figure S1 and IHC of P-S6 is shown in Supplementary Figure S2).

**Table 2:** Molecular characteristics of TNBC PDX models and response to everolimus treatment

PDX	Histology	Neo-adjuvant	TNBC subtype	INPP4B (IHC)	PTEN (IHC)	PAKT (IHC)	P-S6 (IHC)	PIK3CA	AKT1	Response to everolimus (TGI)
**HBCx-2**	IDC	no	Luminal-like (AR+FOXA+)	+	++	+	++	wt	E17K	0%
**HBCx-12A**	IDC	docetaxel	HER2 enriched	lost	lost	+	++	wt	wt	29% (ns)
**HBCx-16**	IDC	no	Basal (KRT5+KRT17+) / HER2 enriched	lost	+	++	+	wt	wt	0%
**HBCx-30**	IDC	no	Basal (KRT5+KRT17+)	lost	lost	-	+	wt	wt	30% (ns)
**HBCx-60**	MBC (spindle)	no	Mixed EGFR+ Mesenchymal (CDH1 -)	lost	+	++	++	H1047K	AMP	27% (ns)
**HBCx-39**	IDC	EC + docetaxel	Basal (KRT5+KRT17+)/HER2 enriched	lost	+	-	+	wt	wt	42% (p=0.057)
**HBCx-31**	IDC	no	Luminal-like (AR+FOXA1+)	+	+	++	+	wt	E17K	41% (p<0.05)
**HBCx-66**	IDC and MBC (spindle)	FEC + docetaxel	Basal (KRT17+)	lost	lost	++	++	wt	wt	50% (p<0.001)
**HBCx-69**	MBC (chondroid)	FEC + docetaxel	Mixed basal (KRT5+KRT17+)/Mesenchymal (CDH1-)	lost	lost	+	+	wt	wt	58% (p<0.05)
**HBCx-10**	IDC	no	HER2 enriched	lost	lost	+	+	wt	wt	60% (p<0.005)
**HBCx-51**	IDC	no	Basal (KRT5) / HER2-enriched	lost	+	-	++	wt	wt	72% (p<0.001)
**HBCx-4B**	IDC	no	Basal (KRT5+ KRT17+)	lost	lost	++	++	E545K	wt	70% (p<0.005)
**HBCx-52**	IDC	no	Luminal-like AR+ FOXA1+	+	+	+	++	wt	E17K	72% (p<0.005)
**HBCx-24**	IDC	no	Basal (KRT5+)	+	lost	++	+	wt	wt	73% (p<0.05)
**HBCx-63**	IDC	FEC docetaxel	unclassified	lost	lost	+	++	wt	wt	80% (p<0.005)

IDC: infiltrating ductal carcinoma; MBC: metaplastic breast carcinoma; FEC: fluorouracil (5-FU), epirubicin, cyclophosphamide; EC: epirubicin + cyclophosphamide; TGI=tumor growth inhibition; ns: not significant. Wt: wild-type. AMP: amplification. Ns: not significant

**Figure 2:**
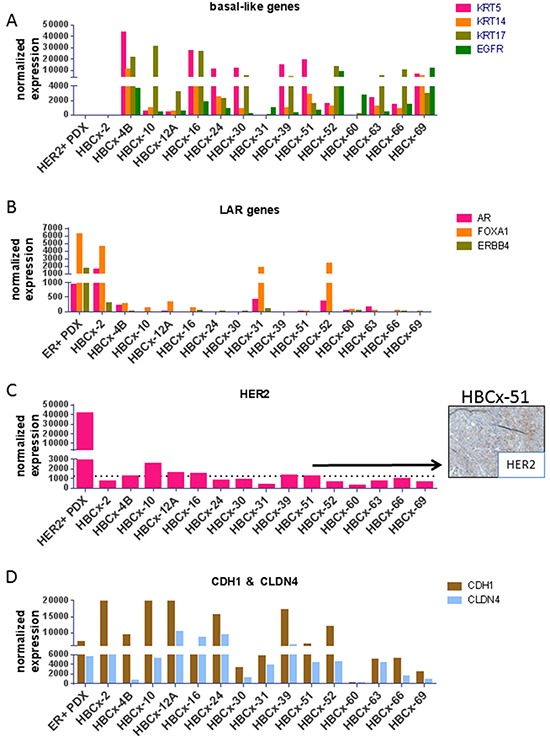
RT-PCR expression analysis of selected genes differentially expressed in molecular subtypes of TNBC **A.** Expression of the basal-like genes *KRT5, KRT14, KRT17* and *EGFR* genes. **B.** Expression of the LAR genes *AR, FOXA1* and *ERBB4*. **C.** Expression of the *HER2* gene and IHC analysis of HER2 expression in the HBCx-51 PDX (classified as HER2 2+). **D.** Expression of *CDH1* and *CLDN4* genes, known to be low or null in the mesenchymal subtypes. As positive controls, an ER+ and HER2+ PDX are shown in the LAR genes and HER2 graphs, respectively.

The molecular TNBC subtype of the 15 PDX was determined based on mRNA expression of a set genes (*KRT5, KRT14, KRT17, EGFR, AR, FOXA1, ERBB4, HER2, CDH1, CLDN4)* known to be differentially expressed in basal-like, luminal-AR (LAR), HER2+ enriched and mesenchymal-like/claudin-low triple-negative tumors [9–11]. Seven PDX showed high expression of *KRT5* indicating a basal-like phenotype and 2 of them also expressed high levels of EGFR (HBCx-4B and HBCx-69) (Figure 2). The HBCx-2, HBCx-31 and HBCx-52 PDX, all carrying the E17K *AKT1* mutation, showed expression of androgen receptor (*AR*) and *FOXA1* genes and low/null expression of *KRT5* suggesting a LAR phenotype (luminal-AR). Five PDX showed expression of HER2. The expression level of HER2 in the HBCx-51 tumor was taken as cut off for positivity, the patient's tumor of this PDX was classified as HER2 2+ in IHC based on international guidelines on HER2 scores (and without gene amplification) (Figure 2) [12]. The HBCx-12A was the only PDX clearly HER2-enriched showing expression of HER2 combined with absence of basal and luminal markers (absence of HER2 amplification was confirmed by comparative genomic hybridization array). Moreover, the analyses revealed a mixed epithelial (EGFR+) and mesenchymal (CDH1 null) phenotype for the HBCx-60 (established from metaplastic breast cancer with a spindle cell component). To confirm RT-PCR results on TNBC subtype classification, the expression of AR, FOXA1, KRT5, KRT17, CDH1 and VIM was analyzed by IHC analysis in the 15 PDX models (Supplementary Figure S3-S5). Overall, gene and protein expression levels were correlated for all investigated markers. Two of the 3 LAR models were KRT5 and KRT17 negative at the protein level, Vimentin was highly expressed in 2 out of 3 metaplastic breast cancer, which also showed loss of E-cadherin expression (Supplementary Figure S5).

**Figure 3:**
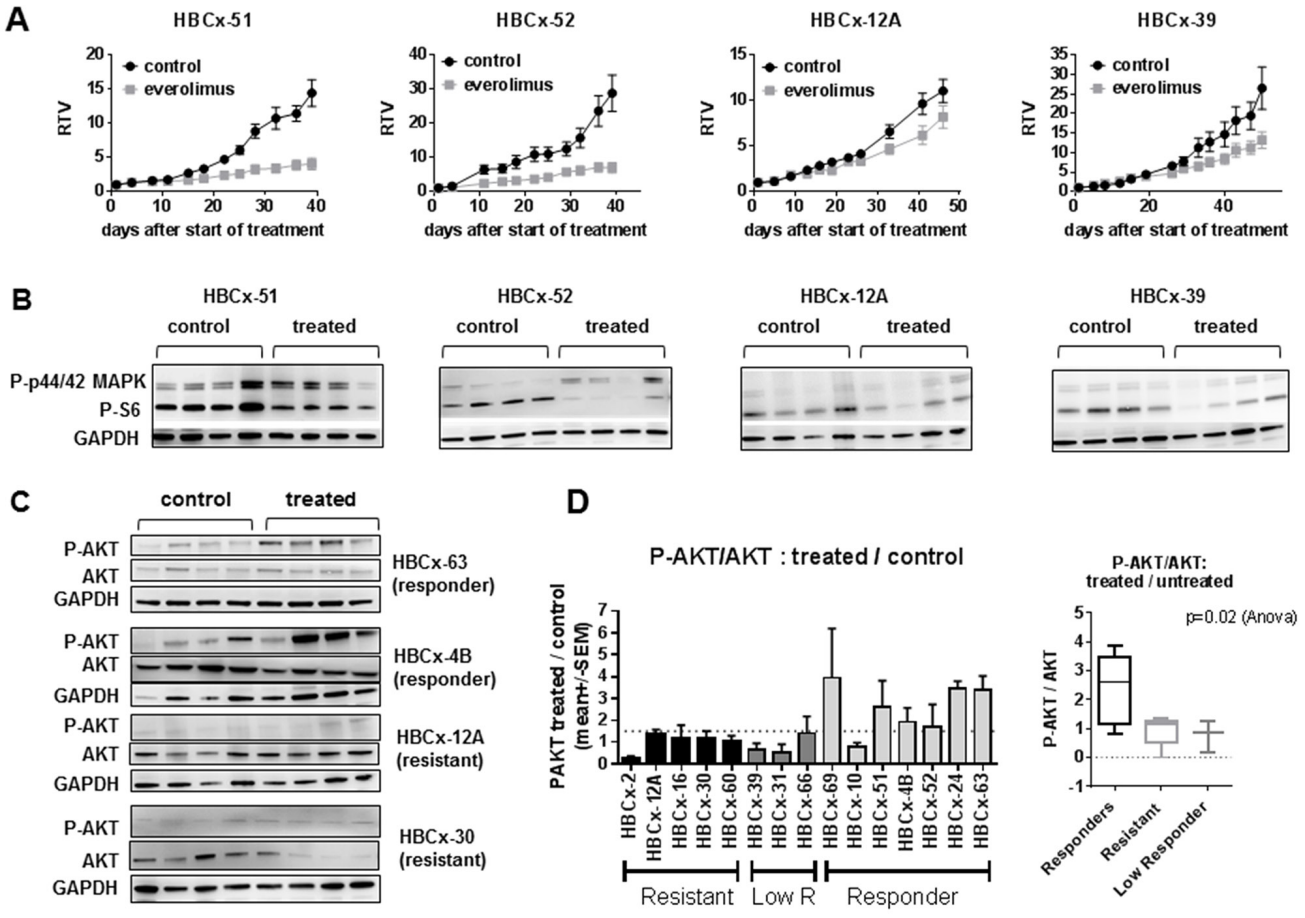
*In vivo* activity of everolimus and feedback activation of AKT **A.** *In vivo* response to everolimus in two responder models (HBCx-51 and HBCx-52), one low responder (HBCx-39) and one resistant (HBCx-12A) PDX. RTV= Relative tumor volume. Mean of RTV +/− SD (n=8). **B.** Western blot analysis of P-S6 and P-p44/42 MAPK in the 4 PDX at the end of experiments (n=4). **C.** Western blot analysis of AKT phosphorylation (Ser473) in 2 responder (HBCx-63 and HBCx-4B) and 2 resistant (HBCx-12A and HBCx-30) PDX models in untreated and everolimus-treated tumors. N=4 xenografts. Western blot of GAPDH in HBCx-12A samples is the same in Figure 3B and 3C. **D.** Ratio of treated/untreated P-AKT/AKT in the 15 PDX models according to everolimus response. P-AKT and AKT were normalized on GAPDH expression. The ratio represents the mean of P-AKT/AKT in 4 treated mice / mean of P-AKT/AKT in 4 untreated mice.

To determine the response of these PDX models to an mTOR inhibitor, we treated them with everolimus as single agent, given at 2.5 mg/kg 3/week, a dose well tolerated and highly efficient in PDX models of ER+ breast cancer [13]. Treatment by everolimus resulted in a tumor growth inhibition (TGI) greater than 50% in 7 out of 15 models, 3 models showed moderate/intermediate response with a TGI between 41% and 50% and 5 PDX models did not respond to everolimus treatment (Table 2). The *in vivo* response of two responders (HBCx-51 and HBCx-52), one low-responder (HBCx-39) and one resistant (HBCx-12A) PDX models are illustrated in Figure 3A. The PDX response to conventional chemotherapies such as AC (doxorubicin and cyclophosphamide) and docetaxel has been previously published [14, 15]. Interestingly, response to everolimus was observed even in PDX models resistant or low responder to chemotherapy (Supplementary Table S1) and in 2 PDX models established from residual tumors after neo-adjuvant chemotherapy (HBCx-63 and HBCx-69). Response to everolimus was not restricted to a particular TNBC subtype: basal, LAR and HER2-enriched were present in both responder and resistant/low responder groups (Table 2).

Then we analyzed the association between PI3K markers and everolimus response. Loss of PTEN was more frequent in responder models (5 out of 7) compared to resistant/low responder models (3 out of 8), however the association between PTEN loss and everolimus response was not statistically significant (p=0.31, Fisher's exact test). Loss of INPP4B occurred in 5 out of 7 responder PDX and 6 out of 8 resistant/low responder PDX. Concomitant loss of PTEN and INPP4B occurred in 4 responder and 3 resistant/low responder tumors (p=0.61, Fisher's exact test).

*PIK3CA* and *AKT1* mutations and baseline phosphorylation level of AKT were not sufficient to predict response either. We also analyzed expression of TSC2 (Tuberous Sclerosis Complex 2) and LKB1 (liver kinase B1) by RT-PCR and western blot. Both are tumor suppressor genes that negatively regulate mTOR [16, 17]. Loss of TSC2 was associated to everolimus response in PDX models of hepatocellular carcinoma [18] and breast cancer patients with low level of LKB1 protein derived greater benefit from everolimus in combination with tamoxifen in the TAMRAD trial [19]. In our panel of PDX, we did not find any correlation between everolimus response and low expression or loss of expression of TSC2 and LKB1 (data not shown). Finally, we investigated the TP53 gene status: loss-of function mutations were found in 5 responder PDX (HBCx-69, HBCx-10, HBCx-4B, HBCx-24 and HBCx-63), 3 low responder (HBCx-39, HBCx-31, HBCx-66) and 2 resistant PDX (HBCx-2 and HBCx-30) (data not shown). There was no relationship between TP53 status and response to everolimus.

In summary, these data show that in 7 out of 15 TNBC everolimus inhibits tumor growth with a TGI > 50%, and that this response occurs in different TNBC subtypes (LAR, basal, mesenchymal or HER2-enriched). PI3K pathway markers at baseline are not sufficient to predict everolimus response in this cohort of tumors.

### Feedback reactivation of AKT in treated tumors is associated to response to everolimus

To assess whether everolimus efficacy was correlated to mTOR inhibition, we analyzed the expression of P-S6, an everolimus downstream biomarker [20], in both responder and resistant models. We performed analysis of P-S6 expression by western blot including 4 mice of treated and untreated tumors. Figure 3B shows western blot analysis of P-S6 in two responder (HBCx-51 and HBCx-52), one low responder (HBCx-39) and one resistant (HBCx-12A) PDX. We observed a significant reduction of P-S6/S6 ratio in treated tumors of 9 out of 15 PDX models (Supplementary Figure S6A). Inhibition occurred in 6/7 responder tumors and in 3 out of 8 resistant/low responder tumors. In 3 PDX models (HBCx-16, HBCx-31, HBCx-66) there was a decrease in P-S6/S6 ratio, not significant due to heterogeneity between the 4 replicates.

The association between inhibition of S6 phosphorylation and everolimus response was not statistically significant (p=0.11, Fisher's exact test), but it indicates that in some tumors everolimus inhibits mTOR function without affecting cell growth. To determine whether the lack of P-S6 inhibition in resistant tumors was due to a failure of everolimus treatment to inhibit mTOR and p70S6K kinases, we further analyzed mTOR signaling in 2 everolimus-resistant models: HBCx-2, with no inhibition of P-S6, and HBCx-16, with a trend toward inhibition of P-S6 that was not statistically significant (Supplementary Figure S6A and S6B). Results, obtained from the analysis of 4 replicats/group/model, show that in HBCx-2, the ratio of P-mTOR/mTOR and P-p70S6K/p70KS6K were not decreased by everolimus treatment, while in HBCx-16 the decrease was statistically significant (Supplementary Figure S6C).

We next asked the question whether the response or resistance to everolimus were associated to activation of compensatory pathways or feedback loops. Among these are the re-activation of PI3 kinase and mitogen-activated protein kinase (MAPK) signaling pathways in the setting of mTORC1-specific inhibitors, caused by the relief of S6K- mediated repression of IRS1 [21, 22]. We analyzed the phosphorylation levels of AKT and P-44/42 MAPK in untreated and treated tumors of both responder and resistant models, harvest at the end of the *in vivo* treatment. Western blot analysis of P-AKT (ser473) in control and everolimus-treated tumors from two resistant (HBCx-12A and HBCx-30) and two responder (HBCx-63 and HBCx-4B) PDX are shown in Figure 3C. AKT appeared phosphorylated in treated tumors of everolimus-responder xenografts. The ratio of P-AKT in treated versus untreated tumors was higher or equal to 1.5 in 6 out 7 responding models, while it was lower than 1.5 in resistant or low responder models (Figure 3D). In the panel of 15 PDX, the P-AKT/AKT ratio in treated versus untreated tumors was higher in the responder PDX models when compared to resistant and low responder PDX (p=0.02, Anova test).

Similarly, western blot analyses showed increase in P-44/42 MAPK levels in treated mice of responder models, indicating a trend of MAP kinase pathway post-treatment activation in responder models compared to resistant models (Supplementary Figure S7).

Finally, we tested whether targeting both mTORC1 and mTORC2 complexes results in improved anti-tumor activity. The efficacy of the dual mTORC1/mTORC2 inhibitor AZD2014 [23] was tested in two PDX models: HBCx-63 (everolimus-responder) and HBCx-16 (everolimus-resistant). The HBCx-63 PDX was established from a residual breast cancer after neo-adjuvant chemotherapy (Table 2).

**Figure 4:**
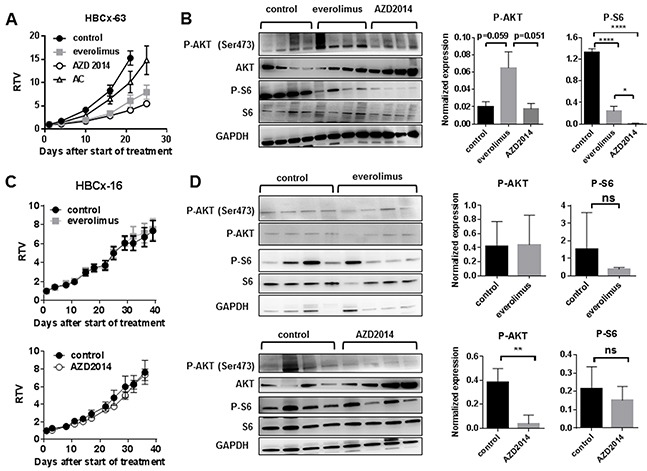
Treatment of HBCx-63 and HBCx-16 by the dual mTORC1/mTORC2 inhibitor AZD2014 as compared to everolimus **A.** *In vivo* response to everolimus, AZD2014 and AC in the HBCx-63 PDX model. AC: Adriamycin + cyclophosphamide. **B.** Western blot analysis of P-AKT (Ser473), AKT, P-S6, S6 and GAPDH expression in treated HBCx-63 tumors (n=4). Statistical analysis of P-AKT and P-S6 expression differences between control, everolimus and AZD2014 treated groups was performed on normalized expression values by unpaired t-test. *<0.05; ****<0.0001. **C.** *In vivo* response of HBCx-16 to everolimus and AZD2014. **D.** Western blot analysis of P-AKT (Ser473), AKT, P-S6, S6 and GAPDH expression in treated HBCx-16 tumors (n=4). Statistical analysis of P-AKT and P-S6 expression differences between control, everolimus and AZD2014 treated groups was performed by unpaired t-test. **<0.005; ns=not significant.

As AZD2014 inhibits both mTORC1 and mTORC2 substrates, in contrast to everolimus that only inhibits mTORC1 substrates, post-treatment upregulation of phosphorylation of AKT does not occur [23]. In the HBCx-63 PDX, treatment by AZD2014, given at the dose of 15mg/kg/day, resulted in tumor growth inhibition of 71%, similar to the effect of everolimus in this experiment (69%) (Figure 4A). This tumor is resistant to the combination of doxorubicin and cyclophosphamide (AC). Analysis of tumors at the end of experiment confirmed increased level of P-AKT in the everolimus-treated tumors, while no increase was detected in the AZD2014-treated tumors, indicating that mTORC2 activity was inhibited (Figure 4B). Phosphorylation of S6 was decreased in both treated groups, with a greater inhibition for the AZD2014 treated tumors (Figure 4B). The HBCx-16 PDX model, resistant to everolimus treatment, was equally resistant to AZD2014 (Figure 4C). No significant decrease of P-S6 was found in both everolimus and AZD2014-treated xenografts (Figure 4D). Phosphorylation of AKT, unchanged in everolimus-treated tumors, was decreased by AZD2014 treatment (Figure 4D), indicating inhibition of mTORC2 activity.

In summary, these results indicate that everolimus-induced AKT re-activation is more pronounced in everolimus-responder tumors. *In vivo* response to the dual mTOR inhibitor AZD2014, tested in 2 PDX models, was identical to everolimus response, in spite of inhibition of mTORC2 activity.

## DISCUSSION

In this study we analyzed the activation status of PI3K pathway in a large panel of PDX of breast cancer previously described [14, 24, 25]. We found that loss of the tumor suppressors PTEN and INPP4B occurred with high frequencies in TNBC PDX, while PIK3CA and AKT1 mutations were rare. These findings are consistent with the data described in patients’ tumors: Saal et al. showed that PTEN loss is more common in ER- tumors and is mutually exclusive with PIK3CA mutations [26]. INPP4B loss was found in more than 80% of TNBC PDX and was not mutually exclusive with PTEN loss, as 40% of PDX models displayed a concomitant loss of PTEN and INPP4B. These results are similar to what was published by Fedele et al., who showed that INPP4B protein expression loss was associated with high clinical grade and loss of hormone receptors and occurred most commonly in aggressive basal-like breast carcinomas, including PTEN-null tumors [6].

The mTOR inhibitor everolimus has been recently approved for the treatment of advanced ER+ breast cancer [7], and several trials are currently evaluating the efficacy of mTOR inhibitors in the TNBC subtype [27]. *In vitro*, everolimus has an anti-tumor activity in basal-like breast cancer cell lines [28]. In this study, we evaluated the activity of everolimus in 15 PDX models of TNBC of different phenotypes and with different expression level of PTEN and INPP4B tumor suppressors.

We found an important reduction of tumor growth superior of 50% in 7 out of 15 PDX, although everolimus did not induce tumor regressions, this result is consistent with a recent work showing that everolimus inhibits tumor growth in PDX models of TNBC without tumor eradication [29].

The identification of TNBC tumor types that may respond to mTOR inhibitors remains a major issue. Although the number of PDX used in this study is limited, it represents a heterogeneous panel in term of TNBC subtype, tumor histology, activation of PI3K pathway and response to chemotherapies. Here we show that tumor response to everolimus is not associated with a specific TNBC subtype, as basal-like, LAR, mesenchymal and HER2-enriched tumors were present in both responder and resistant groups. In addition, everolimus showed anti-tumor activity in two PDX established from metaplastic breast carcinomas, a rare histological subtype of breast cancer with poor prognosis, characterized by a mixed epithelial/mesenchymal histology and frequent molecular aberrations in the PI3K pathway [30].

Since mTOR is expressed in tumor tissues and healthy organs, the sensitivity or resistance to mTOR inhibitors cannot be predicted upon the presence of the target. For this reason, the overall activation of the PI3K/AKT/mTOR pathway has been proposed to identify tumor types that could be sensitive to mTOR inhibitors. In human cell lines, *PIK3CA/PTEN* genomic aberrations and high P-AKT levels are associated with rapamycin sensitivity *in vitro* [31]. However, thus far, parameters reflecting activation of the PI3K/AKT/ mTOR pathway have failed to predict *in vivo* sensitivity to rapalogs in most tumor types. The main intrinsic parameters of this pathway that have been assessed in tumor models as biomarkers of sensitivity, alone or in combination, are the loss of PTEN function, AKT phosphorylation, and *PIK3CA* mutations. Our results indicate that loss of PTEN, INPP4B or both are not sufficient to confer sensibility to everolimus (PTEN loss occurred more frequently in responder models but this association was not statistically significant). Published data on PTEN as a biomarker are controversial. Neshat et al. reported the enhanced sensitivity of PTEN-deficient tumors to the inhibition of mTOR [32]. This has been confirmed *in vivo* by using human prostate xenografts, against which temsirolimus displayed limited activity when PTEN was functional [33]. By contrast, in bladder cancer PTEN deficiency is associated with reduced sensitivity to mTOR inhibitor [34]. Finally, Yunokawa did not found a correlation between PTEN loss and response to everolimus in TNBC cell lines [28].

Three out of 5 resistant PDX did not show a significant decrease in P-S6 after everolimus treatment: in one case P-S6 was not expressed at the basal level (HBCx-30), in HBCx-16 we could detect P-mTOR and P-p70S6K inhibition with a trend toward P-S6 inhibition and in the HBCx-2 PDX mTOR and p70S6K kinases were not inhibited. These results suggest that everolimus resistance and lack of P-S6 inhibition in these tumors could be due to different reasons, including lack of target expression (HBCx-30), failure of everolimus to inhibit mTOR (HBCx-2), or incomplete mTOR and p70S6K inhibition that might be not sufficient to have antiproliferative activity.

In the 15 models tested, response to everolimus was not associated to intrinsic AKT phosphorylation but rather to treatment-induced phosphorylation of AKT. Inhibition of mTOR kinase relieves feedback inhibition of receptor tyrosine kinases, leading to subsequent PI3K activation and rephosphorylation of AKT sufficient to reactivate AKT activity and signaling [35]. The feedback loop activation of AKT has been proposed as mechanism of everolimus resistance, however we show here that treatment-induced phosphorylation of AKT occurs in everolimus responder models and not in resistant PDX. Hence high levels of pathway re-activation may merely reflect dependence of tumors on the pathway, as opposed to a resistance mechanism.

The same observation has been reported by Meric-Bernstam *et al.*, who showed that rapamycin treatment is associated with increased p-AKT in sensitive breast cancer models *in vitro* and *in vivo,* indicating that treatment-associated increase in P-AKT is not a marker of resistance but rather of sensitivity [31]. By contrast, Breuleux et al. did not find a correlation between everolimus sensitivity in cancer cell lines and induction of AKT phosphorylation following treatment [36]. However, comparison between *in vitro* and *in vivo* studies should be interpreted cautiously as the experimental conditions differ considerably: *in vitro,* everolimus response and P-AKT expression were analyzed after an exposure of 24h, while in PDX models tumors were analyzed after 4 or 5 weeks of treatment. In addition, those studies included cell lines from different cancer types that might have different mechanisms of PI3K/AKT/mTOR activation.

Another feedback loop associated to mTOR inhibition has been discovered by Corracedo et al., who found that mTORC1 inhibition can activate the MAPK pathway *in vitro*, in mouse models or in human tumor samples [22]. As for the PI3K/AKT feedback loop, our results show a trend to activation of MAPK signaling occurs in everolimus-responder models. The lack of reactivation of MAPK and PI3K pathway activation in everolimus resistant models suggest that these feedback mechanisms are not associated to the intrinsic resistance to everolimus in these tumors, which could be linked to other mechanism [37, 38]. Additional experiments will be necessary to determine whether the combination of mTOR and MEK inhibitors could increase the tumor response of TNBC. Concomitant inhibition of MAPK and PI3K/mTOR signaling was shown to be synergistic in mouse models of breast cancer [39]. Preclinical studies in other tumor types have shown that dual inhibition of both the PI3K and MEK/ERK pathways with a number of different small molecule kinase inhibitors leads to greater growth inhibition than single pathway inhibition [40–42]. However, since the PI3K/mTOR and MAPK pathways play central role in normal tissues, their simultaneous targeting in patients could result in severe adverse effects. Several trials are ongoing to test the safety and efficacy of such dual inhibition [43].

Treatment of an everolimus-responder and chemo-resistant PDX with an mTORC1/mTORC2 inhibitor (AZD2014) resulted in tumor growth inhibition, with a strong inhibition of P-S6 and an expected lack of P-AKT reactivation due to mTORC2 inhibition [23]. This preliminary result suggests that targeting mTORC1/2 activities could be efficacious not only in ER+ breast tumors but also in aggressive chemo-resistant TNBC. It also suggests that, at least in this PDX model, inhibiting both mTORC1 and mTORC2 activities and post-treatment P-AKT reactivation does not necessarily results in tumor regression. Similarly, when administered to the everolimus-resistant PDX HBCx-16, AZD2014 treatment did not result in increased anti-tumor activity and P-S6 was not significantly inhibited after 5 weeks of treatment. In this tumor lack of both everolimus and AZD2014 efficacy may be a consequence of an inefficient mTORC1 targeting. Additional experiments will be necessary to analyze in depth the mechanisms of resistance to mTOR inhibitors and to further characterize the preclinical activity of AZD2014 in TNBC.

In conclusion, we have analyzed a large panel of PDX models of breast cancer for PI3K pathway activation. This panel represents a useful tool for preclinical testing of PI3K/AKT/mTOR pathway inhibitors. By testing 15 PDX models, representing different histology and phenotypic subtypes of TNBC, we have identified a subgroup of tumors that respond to mTOR inhibition. Although these results need to be confirmed in larger cohorts of tumors, post-treatment activation of AKT could be a valuable biomarker for early monitoring of response to everolimus in post-treatment biopsies.

## MATERIALS AND METHODS

### Patient-derived xenografts

Female Swiss nude mice, 10-week old, were purchased from Charles River (Les Arbresles, France) and maintained under specific pathogen-free conditions. Their care and housing were in accordance with institutional guidelines as put forth by the French Ethical Committee, as previously detailed [44]. Sixty-seven breast cancer PDX, including 42 TNBC, 17 ER+ and 8 HER2+, were used in this study [14, 44]. They were all established from primary surgical specimens with patient informed consent, as described elsewhere [14, 44]. The ER+ PDX HBCx-3, HBCx-21, HBCx-22 and HBCx-34 have been described by Cottu et al. [24, 45], HBCx-1 to HBCx-63 have been published by Marangoni et al. [14], Reyal et al. [25] and Hatem et al. [44]. HBCx-64 to HBCx-69, HBCx-73, HBCx-81, HBCx-86, HBCx-87, HBCx-90, HBCx-92, HBCx-95, HBCx-100, HBCx-101, HBCx-106 and HBCx-108 have not been previously published.

The 15 PDX chosen for *in vivo* experiments were established from primary breast tumors with the exception of HBCx-4B, established from a lymph node metastasis. HBCx-12A, HBCx-39, HBCx-66 and HBCx-69 PDX were established from patients with residual tumors after neo-adjuvant chemotherapy. Tumor fragments were grafted into the inter-scapular fat pad of female nude mice and maintained through *in vivo* passages as previously described [14]. The ER+, HER2+ and triple-negative status was confirmed in PDX models by IHC analysis as detailed previously [14, 25].

Everolimus was purchased from Novartis and administered orally at a dose of 2,5mg/kg 3xweek [45]. AZD2014 was provided by Astrazeneca and was administered orally at a dose of 15 mg/kg/day [23]. Everolimus was formulated in distillated water and AZD2014 in MCT (0.5% methylcellulose/0.2% tween 80).

Tumor growth inhibition (TGI) of treated tumors versus controls (non-treated tumors) was calculated as the ratio of the mean RTV (relative tumor volume) in treated group to the mean RTV in the control group at the end of the experiment [14]. A two-tailed student t-test was used for statistical analysis of tumor growth inhibition. PDX models were considered responder to everolimus when TGI was superior to 50%, low responder when TGI was comprised between 40% and 50%, and resistant, when TGI was inferior to 40% or when tumor growth was not significantly altered by the treatment [14].

### Mutation screening

Mutations of *PIK3CA* (exons 9 and 20) and *AKT1* (exon 4) were detected by sequencing of cDNA fragments obtained by RT-PCR amplification. Details of the primers and PCR conditions are available on request. The amplified products were sequenced with the BigDye Terminator kit on an ABI Prism 3130 automatic DNA sequencer (Applied Biosystems, Courtaboeuf, France) with detection sensitivity of 10% mutated cells, and the sequences were compared with the corresponding cDNA reference sequences (PIK3CA NM_006218, AKT1 NM_005163).

### Real time RT-PCR analyses

Total RNA was extracted from breast tumor xenografts samples by using acid-phenol guanidium method as previously described [46]. cDNA synthesis and PCR conditions were also previously described [47].

Quantitative values were obtained from the cycle number (Ct value) at which the increase in the fluorescence signal associated with exponential growth of PCR products started to be detected by the laser detector of the ABI Prism 7900 sequence detection system (Perkin-Elmer Applied Biosystems, Foster City, CA), using PE biosystems analysis software according to the manufacturer's manuals.

Transcripts of the *TBP* gene (Genbank accession NM_003194) encoding the TATA box-binding protein (a component of the DNA-binding protein complex TFIID) were also quantified as an endogenous RNA control. Each sample was normalized on the basis of its *TBP* content. Results, expressed as N-fold differences in Target gene expression relative to the *TBP* gene and termed “N*_Target_*”, were determined as N*_Target_* = 2*^ΔCtsample^*, where the ΔCt value of the sample was determined by subtracting the average Ct value of Target gene from the average Ct value of *TBP* gene.

The N*_Target_* values of the samples were subsequently normalized. The normalization was done to obtain a basal mRNA level (smallest amount of mRNA quantifiable (Ct = 35 with 2.5 ng cDNA)) equal to 1. Target mRNA levels that were totally absent or very low (Ct > 35; detectable but not reliably quantifiable) were scored “0” (non expressed).

The primers for the genes expression analysis were chosen with the assistance of the Oligo 6.0 program (National Biosciences, Plymouth, MN). To avoid amplification of contaminating genomic DNA, one of the two primers was placed at the junction between two exons. Agarose gel electrophoresis was used to verify the specificity of PCR amplicons. The primers sequences used are available upon request.

### IHC

Xenografted tumors were fixed in 10% neutral buffered formalin, paraffin embedded, and hematoxylin–eosin stained. The majority of PDX models were included in duplicates or triplicates in 4 different Tissue Micro Arrays (TMA): two tissue cores per tumor were included in the TMA.

Immunostaining were performed in a Discovery XT Platform (Ventana Medical System, Tucson, Arizona, USA, part of Roche Diagnostics) with antigen retrieving using either EDTA buffer, pH 8 (CC1, Ventana Medical System) or citrate buffer 10 mM, pH 6, (CC2, Ventana Medical System). Primary antibodies were monoclonal rabbit antibodies and parallel slides immunostained with rabbit IgG were used as negative controls. Incubation and color development involved anti rabbit multimer secondary antibody (horseradish peroxydase complex) with DAB (3,3′-diaminobenzidine tetrahydrochloride) as substrate (ChromoMap Kit with Anti rabbit OmniMap, Ventana Medical System).

TMAs were analyzed by immunohistochemistry (IHC) for expression of HER2, PTEN, INPP4B, P-S6 and P-AKT (ser474). Immunohistochemistry analysis of AR, CDH1, FOXA1, KRT5, KRT17 and VIM was performed on the 15 TNBC PDX models used for *in vivo* studies.

HER2 (#M3030) and INPP4B (#LS-C137700) rabbit monoclonal antibodies were purchased from Clinisciences (Nanterre, France). AR (#5153), CDH1 (#3195), KRT17 (#12509), P-S6 (#5364), P-TEN (#9559) and P-AKT (Ser 473) (#4060) rabbit antibodies were purchased from Cell Signaling Technology (Ozyme, Montigny Le Bretonneux, France). FOXA1 (#ab23738) and KRT5 (#ab52635) antibodies were purchased from Abcam (Paris, France).

PTEN, INPP4B, P-AKT and P-S6 scores were assigned based on expression intensity: - negative, + weak, ++ strong. PTEN and INPP4B intensities are illustrated in Figure 1.

### Western blot

Proteins were extracted as described previously [13]. Lysates were resolved on 4–12% TGX gels (Bio-Rad®), transferred into nitrocellulose membranes (Bio-Rad®) and immunoblotted with rabbit antibodies against GAPDH (Cell Signaling Technology, #2118), AKT (Cell Signaling Technology, #9272), P-AKT (Ser473) (Cell Signaling Technology, #4058), P-p44/42 MAPK (Cell Signaling Technology, #4370), p44/42 MAPK (Cell Signaling Technology, #9102), S6 (Cell Signaling Technology, #2117), P-S6 (Ser235/236) (Cell Signaling Technology, #2211), mTOR (Abcam, #ab51089), P-mTOR (ser2448) (Abcam, #ab109268), P70S6K (Cell Signaling Technology, #2708), and P-P70S6K (Thr421/Ser424) (Merck Millipore, #04-393).

After washes, membranes were incubated with the appropriate secondary antibodies horseradish peroxidase-conjugated affinity-purified goat anti–rabbit (Jackson ImmunoResearch Laboratories, Inc., Interchim).

Quantification of P-AKT, AKT, P-S6 and S6 was performed by the Multi Gauge software and normalized on GAPDH expression. For each PDX model, the ratio of P-AKT/AKT in everolimus-treated tumors versus control tumors was calculated as follow: mean of P-AKT/AKT in 4 everolimus-treated xenografts / mean of P-AKT/AKT in 4 control xenografts. The same method was used to quantify P-S6/S6. P-p44/42 MAPK was normalized on GAPDH expression as in some PDX total p44/42 MAPK was not detectable. Variance between responder, low responder and resistant xenografts was analyzed by one-way Anova.

## SUPPLEMENTARY FIGURES AND TABLE


