# Supplementary material for: Targeting mTOR pathway inhibits tumor growth in different molecular subtypes of triple-negative breast cancers
Source: Oncotarget. 2016 Jun 21;7(30):48206–19. doi: 10.18632/oncotarget.10195 (PMC5217012; doi:10.18632/oncotarget.10195)
Supplement: Supplementary file 1 [file oncotarget-07-48206-s001.pdf]

Targeting mTOR pathway inhibits tumor growth in different molecular subtypes of triple-negative breast cancers

SUPPLEMENTARY FIGURES AND TABLE

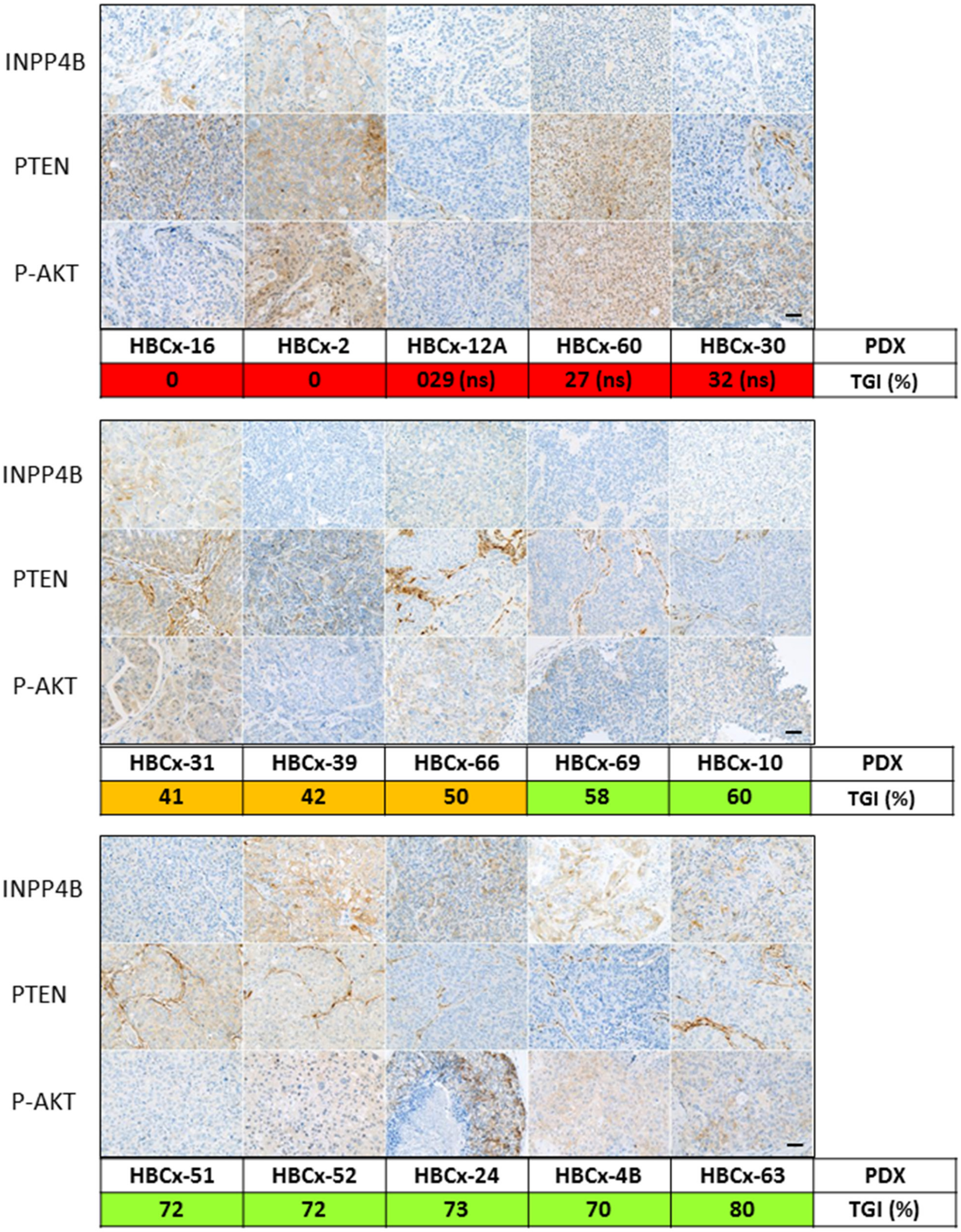

Supplementary Figure S1: Expression of PTEN, INPP4B and P-AKT determined by IHC analysis in the 15 PDX models of Table 2. Scale bar = 50 µm (20x). Response to everolimus is indicated for each PDX model by the TGI (tumor growth inhibition) percentage. Red, orange and green colors indicate resistant, low responder and responder models, respectively.

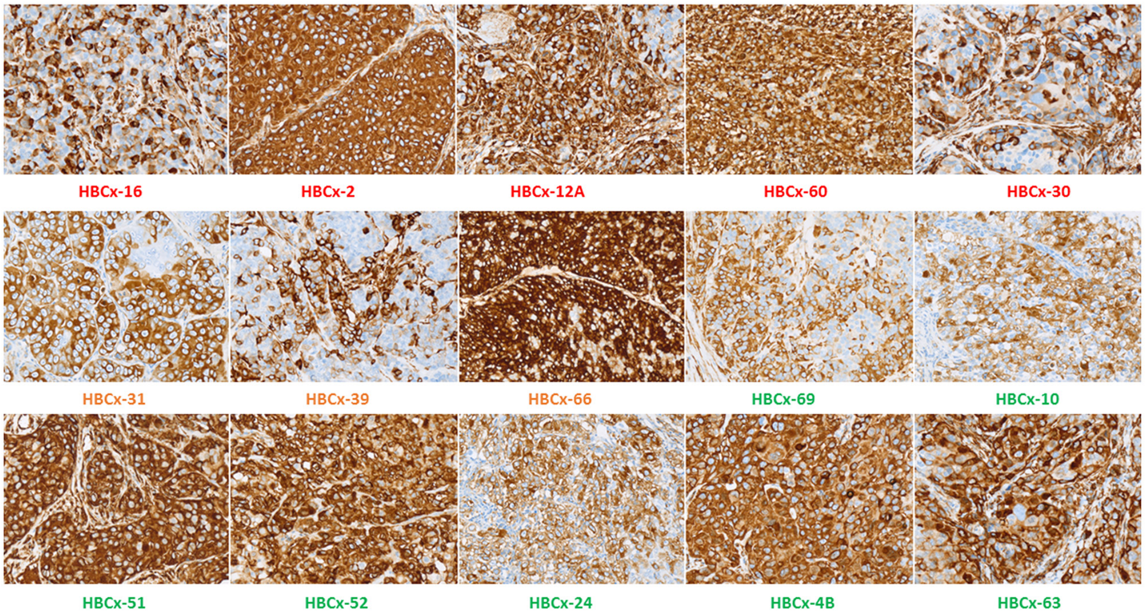

Supplementary Figure S2: Expression of P-S6 determined by IHC in the 15 PDX models. Red: everolimus-resistant PDX, orange: everolimus low responder, green: everolimus-responder PDX.

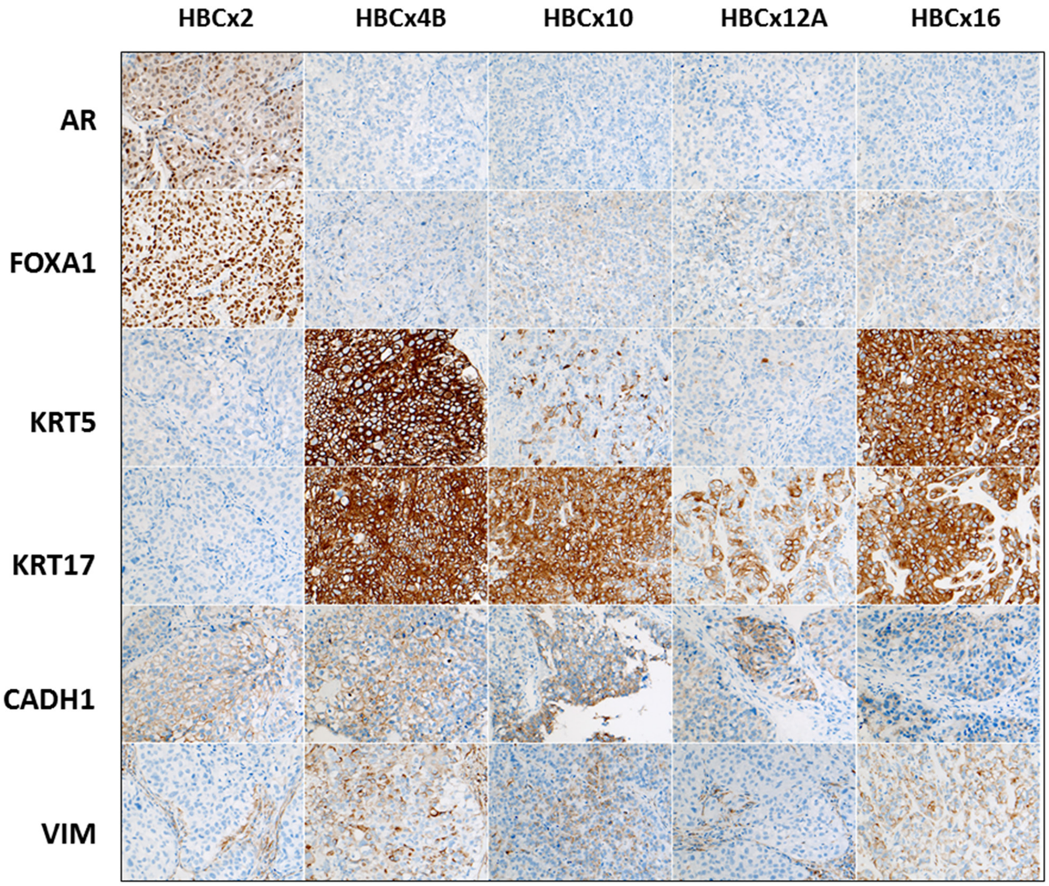

Supplementary Figure S3: Expression of AR, FOXA1, KRT5, KRT17, CDH1 and Vimentin in HBCx-2, HBCx-4B, HBCx-10, HBCx-12A and HBCx-16.

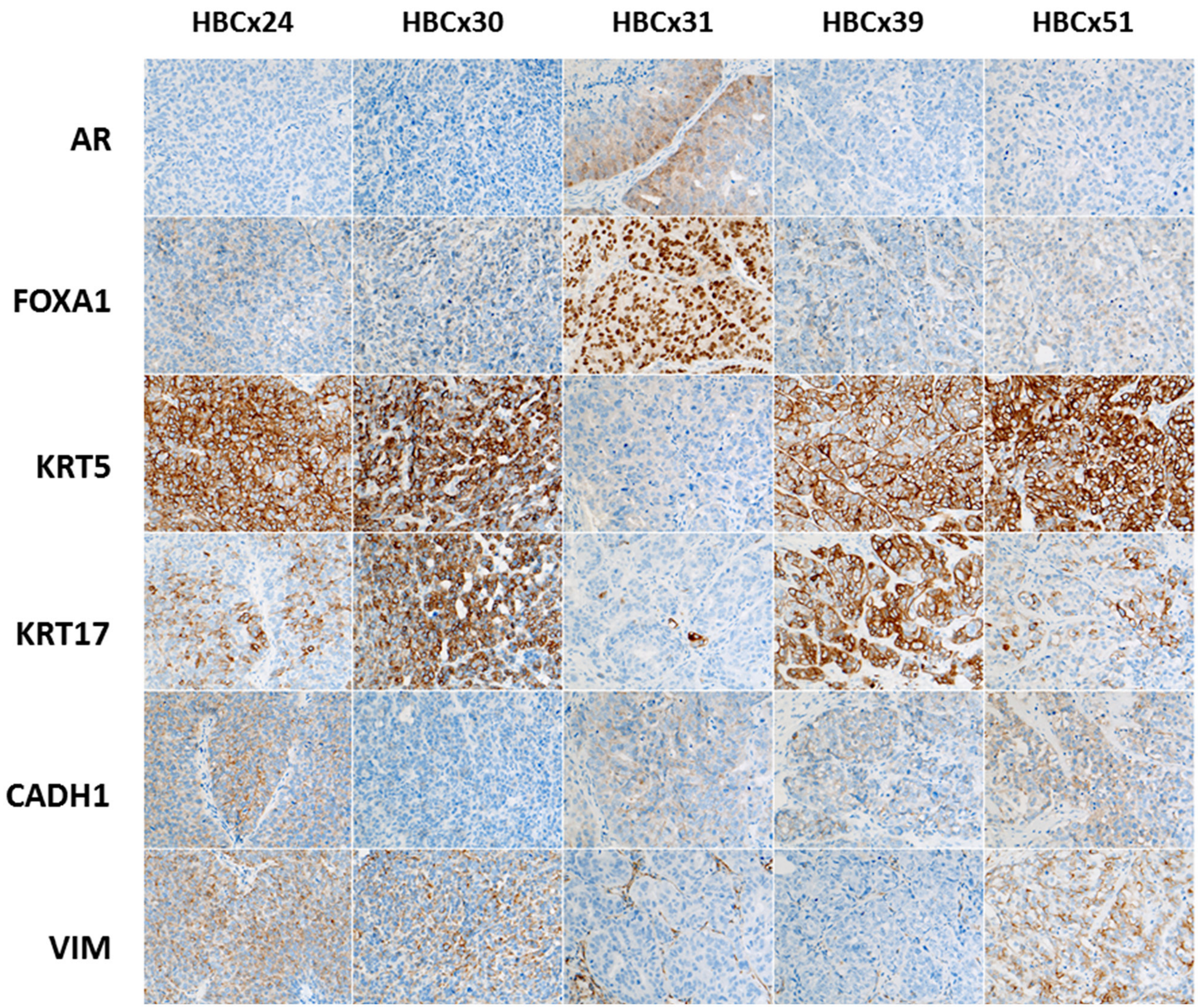

Supplementary Figure S4: Expression of AR, FOXA1, KRT5, KRT17, CDH1 and Vimentin in HBCx-24, HBCx-30, HBCx-31, HBCx-39 and HBCx-51.

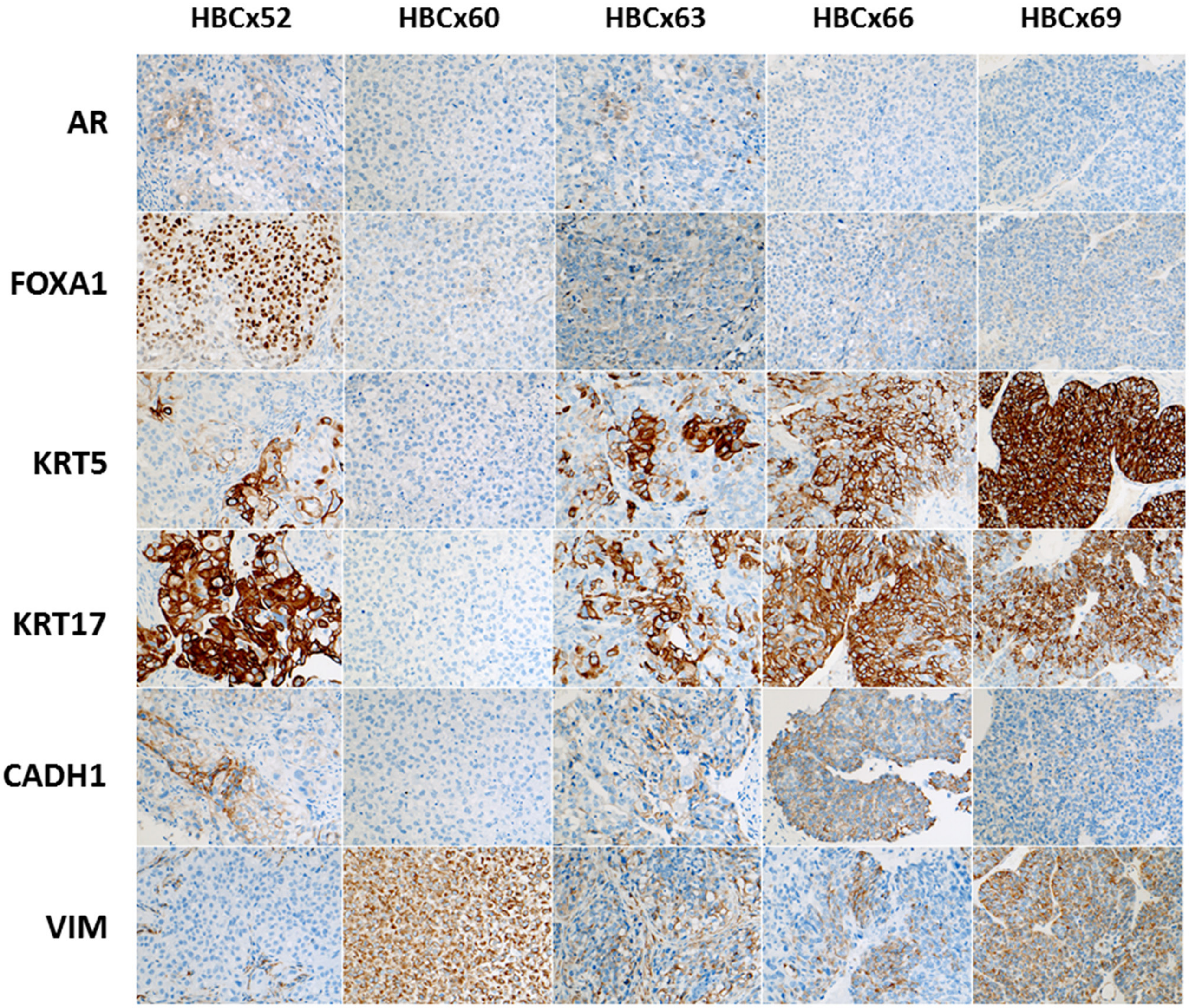

Supplementary Figure S5: Expression of AR, FOXA1, KRT5, KRT17, CDH1 and Vimentin in HBCx-52, HBCx-60, HBCx-63, HBCx-66 and HBCx-69.

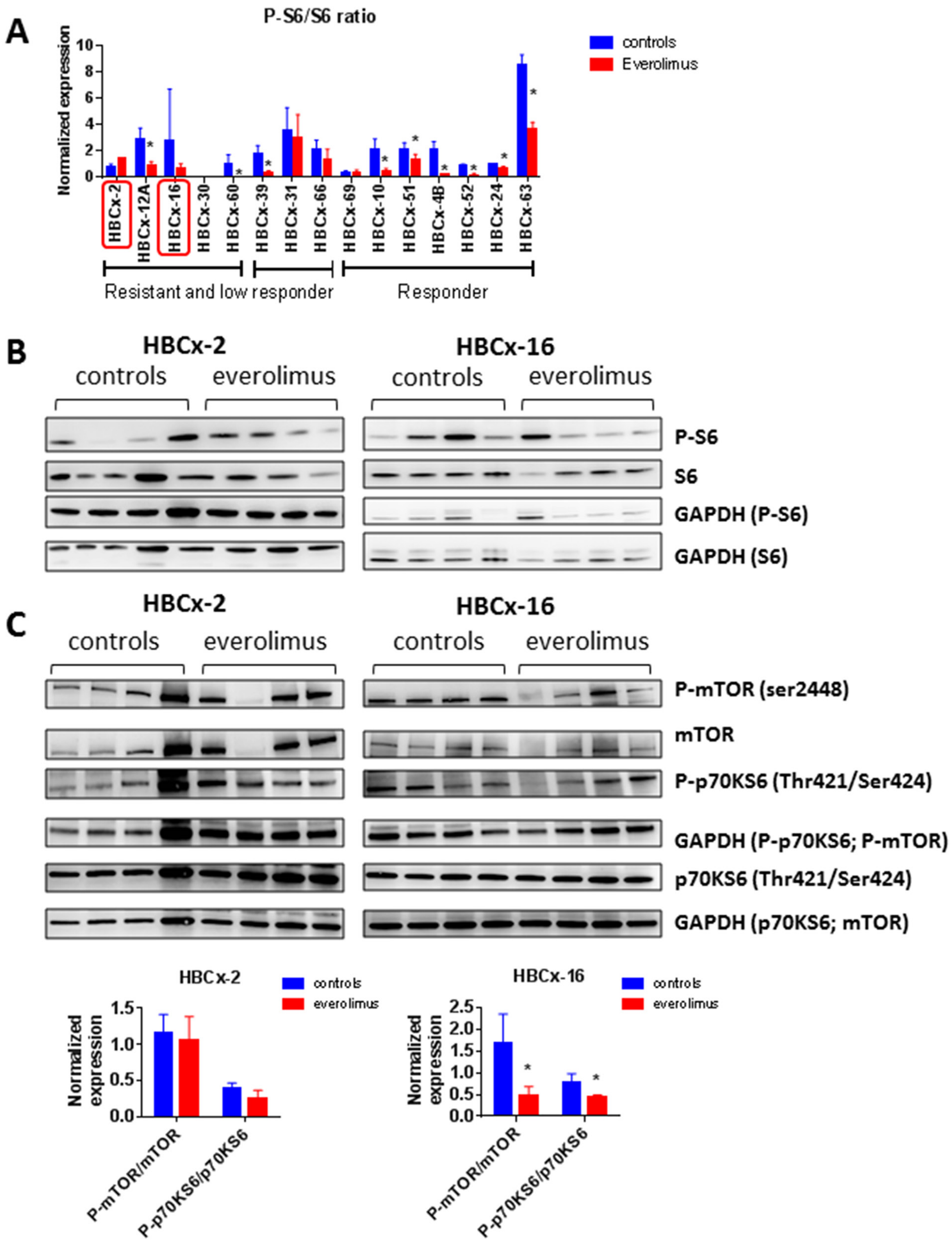

Supplementary Figure S6: A. Ratio of P-S6/S6 in control and treated tumors normalized from Western Blot (mean of 4 xenografts +/- SD). **B.** Western Blot analysis of P-S6 and S6 in 2 everolimus-resistant PDX: HBCx-2 and HBCx-16. **C.** Western Blot analysis of P-mTOR (ser2448), mTOR, P-p70KS6 (Thr421/Ser424) and P-p70KS6. Each protein was normalized against GAPDH and ratio of phosphorylated/ total proteins were calculated with GAPDH-normalized values.

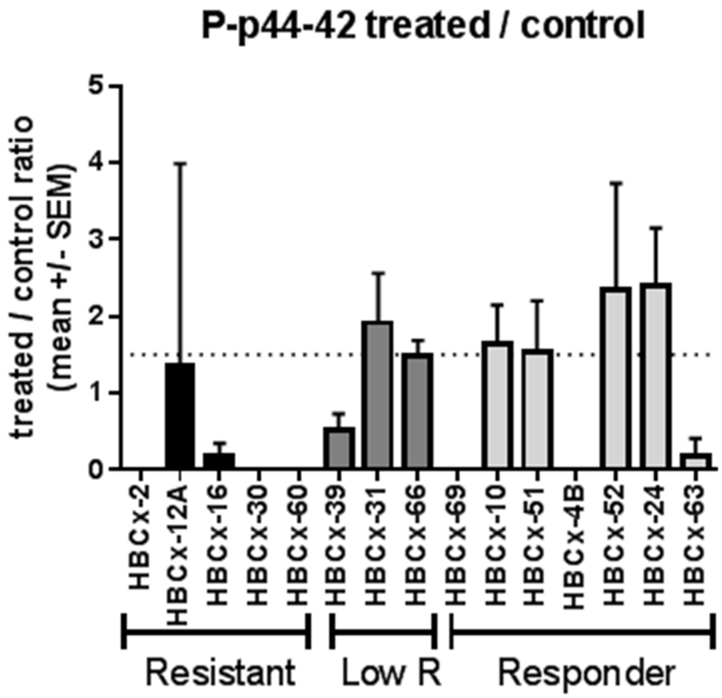

Supplementary Figure S7: Ratio of Phospho-p44/42 MAPK in treated tumors versus control. Normalization of P-p44/42 was performed on GAPDH expression. For each PDX model, the ratio of normalized P-p44/42 in everolimus-treated tumors versus normalized P-p44/42 in control tumors was calculated as follow: mean of P-p44/42 /GAPDH in 4 everolimus-treated xenografts / mean of P-p44/42 /GAPDH in 4 control xenografts.

Supplementary Table S1: Response to everolimus and chemotherapies in the 15 PDX models. AC=doxorubicin + cyclophosphamide

PDX	Response to AC	Response to Docetaxel	Response to Everolimus (TGI)
**HBCx-2**	resistant	resistant	0%
**HBCx-12A**	resistant	resistant	29% (ns)
**HBCx-16**	responder	resistant	0%
**HBCx-30**	responder	low responder	30% (ns)
**HBCx-60**	Not done	Not done	27% (ns)
**HBCx-39**	resistant	resistant	42%
**HBCx-31**	responder	responder	41%
**HBCx-66**	responder	resistant	50%
**HBCx-69**	low responder	low responder	58%
**HBCx-10**	responder	resistant	60%
**HBCx-51**	resistant	responder	72%
**HBCx-4B**	resistant	resistant	70%
**HBCx-52**	not done	not done	72%
**HBCx-24**	resistant	responder	73%
**HBCx-63**	resistant	resistant	80%
